# Views of Medical Doctors Regarding the 2013 WHO Adult HIV Treatment Guidelines Indicate Variable Applicability for Routine Patient Monitoring, for Their Family Members and for Themselves, in South-Africa

**DOI:** 10.1371/journal.pone.0145911

**Published:** 2016-01-21

**Authors:** Willem Daniel Francois Venter, Lee Fairlie, Charles Feldman, Peter Cleaton-Jones, Matthew Chersich

**Affiliations:** 1 Wits Reproductive Health and HIV Institute (RHI), Faculty of Health Sciences, University of the Witwatersrand, Johannesburg, South Africa; 2 Division of Pulmonology, Department of Internal Medicine, Charlotte Maxeke Johannesburg Academic Hospital and Faculty of Health Sciences, University of the Witwatersrand, Johannesburg, South Africa; 3 Steve Biko Centre for Bioethics, Faculty of Health Sciences, University of the Witwatersrand, Johannesburg, South Africa; University Hospitals of Leicester, UNITED KINGDOM

## Abstract

South African doctors (n = 211) experienced in antiretroviral therapy use were asked via an online questionnaire about the WHO 2013 adult antiretroviral integrated guidelines, as well as clinical and personal issues, in three hypothetical scenarios: directing the Minister of Health, advising a family member requiring therapy amidst unstable antiretroviral supplies, and where doctors themselves were HIV-positive. Doctors (54%) favoured the 500 cells/μl WHO initiation threshold if advising the Minister; a third recommended retaining the 350 cells/μl threshold used at the time of the survey. However, they favoured a higher initiation threshold for their family member. Doctors were 4.9 fold more likely to initiate modern treatment, irrespective of their CD4 cell count, for themselves than for public-sector patients (95%CI odds ratio = 3.33–7.33; *P*<0.001, although lower if limited to stavudine-containing regimens. Doctors were equally concerned about stavudine-induced lactic acidosis and lipoatrophy. The majority (84%) would use WHO-recommended first-line therapy, with concerns split between tenofovir-induced nephrotoxicity (55%), and efavirenz central nervous system effects (29%). A majority (61%), if HIV-positive, would pay for a pre-initiation resistance test, use influenza-prophylaxis (85%), but not INH-prophylaxis (61%), and treat their cholesterol and blood pressure concerns conventionally (63% and 60%). Over 60% wanted viral loads and creatinine measured six monthly. A third felt CD4 monitoring only necessary if clinically indicated or if virological failure occurred. They would use barrier prevention (83%), but not recommend pre-exposure prophylaxis, if their sexual partner was HIV-negative (68%). A minority would be completely open about their HIV status, but the majority would disclose to their sexual partners, close family and friends. Respondents were overwhelmingly in favour of continued antiretrovirals after breastfeeding. In conclusion, doctors largely supported adult WHO guidelines as public policy, although would initiate treatment at higher CD4 counts for their family and themselves. Resistance to INH-prophylaxis is unexpected and warrants investigation.

## Introduction

In mid-2013, the World Health Organisation (WHO) released antiretroviral treatment (ART) guidelines, raising the initiation CD4 threshold to 500 cells/μl for adults (and regardless of CD4 threshold for pregnancy and during breastfeeding, for WHO stages 3 and 4, for discordant couples, and for people with chronic hepatitis B), further simplifying treatment choices and recommending continuing lifelong ART after birth for pregnant women (so-called option B-plus) [1]. Most low and middle-income countries have since adopted these guidelines, while treatment guidelines in many high-income and some middle-income countries recommend starting ART irrespective of CD4 count [2].

South Africa has both the largest number of patients with HIV infection, as well as the highest absolute number receiving antiretrovirals, in the world [3]. ART is widely available within the over 3000 South African public health facilities, as well as in the private sector. While implementation of some aspects of the earlier WHO guideline recommendations were delayed, the country’s Department of Health has implemented the 2010 WHO first-line treatment recommendation, replacing stavudine (d4T) with tenofovir (TDF), and increasing the ART initiation threshold to 350 cells/μl. However, the country has been challenged by multiple drug stock-outs, with many patients experiencing ART interruptions and substitutions, leading some to call for caution in further raising initiation thresholds, as recommended in the 2013 WHO guidelines [4]. In addition, there are concerns raised for many low and middle-income countries about the implementation of Option B-plus in women after completing breastfeeding, given the high attrition rates in this group [5, 6]. Debates also surround the role of new antiretrovirals, the use of HIV pre-exposure prophylaxis, and prophylaxis against TB and other infections. Monitoring in the context of ART and concerns about long-term cardiovascular and other risks also commonly elicit discussion [7].

We surveyed the attitudes of South African medical doctors (hereafter referred to as ‘doctors’) experienced in the use of ART, towards the 2013 WHO guidelines and related issues regarding monitoring and disclosure. We examined what practitioners felt should be recommended for public-sector programmes, whether this differed from the way they would treat themselves or their families, and whether ART interruptions and substitutions affect attitudes to these recommendations.

## Methods

An e-mail containing an explanation of the study, inclusion criteria, study consent form, and a link to an online response aggregator (SurveyMonkey; www.surveymonkey.com) was sent in late June 2014 directly to approximately 40 ART-experienced doctors known to one of the authors (WDFV), and to just over 1600 doctor members of the Southern African HIV Clinicians Society (http://www.sahivsoc.org). The email further requested the recipient to forward the email to other experienced treating doctors. Medical doctors were eligible to complete the survey if they had treated more than 50 patients in South Africa with ART, and had experience in providing ART for more than a year. The survey, which closed on July 15^th^ 2014, was only completed online and did not require or allow additional steps to return responses by email or post. The semi-structured survey was built around three scenarios. In the first, doctors were asked to imagine themselves as advisors to the government’s Minister of Health, and to offer their views on the adult ART initiation threshold for the next five years. Their recommendation should only pertain to treatment of clinically uncomplicated adults without TB, hepatitis B and not pregnant. They were also asked their opinion on whether to use Option B (“Treat till breastfeeding cessation or after delivery, if formula fed, if CD4 >350 cells/μl, then interrupt ART”), until the next pregnancy or ART becomes necessary for the woman’s own health, or to support the B-plus option, where ART is continued lifelong.

The second scenario assessed attitudes to initiation thresholds in a family member using the public health system. The specific wording in this scenario was as follows: “You have a strong-willed, HIV-positive, antiretroviral-naïve, completely asymptomatic, poor, female family member, who you are very fond of. However, she insists on using the public health sector, and refuses any help from you other than occasional advice”. Two situations were then described, the first with her only having access to a poorly functioning health system within a rural area, “that has regular intermittent drug stock-outs, reports of stavudine and nevirapine being used in place of tenofovir and efavirenz”. This strategy aimed to test the degree to which the quality of the health system influences doctor choices on the CD4 count initiation threshold. In the second case, their opinions were elicited on use of Option B-plus if the family member was pregnant and had access to a well-functioning health system.

The final scenario assessed the doctor’s attitudes to past (stavudine-based) and current treatments for themselves (‘modern treatment’), in the event of them acquiring HIV infection. We asked about how best to monitor their treatment outcomes and safety after having received ART for more than a year, assuming that they were asymptomatic after their sixth month of ART and had an undetectable annual viral load. Open-ended questions were used to explore ART drug choices that were inconsistent with WHO 2013 recommendations. Respondents were also asked about disclosure of their HIV status, and the use of barriers methods and pre-exposure prophylaxis for an HIV-negative partner. Those who were already HIV positive were requested to assume it was a new diagnosis, for the purposes of the questionnaire. GraphPad QuickCalcs was used for determining the distribution of the participant’s responses and for detecting associations between independent groups of doctors, such as between those working in the public or private sector using Fisher’s Exact test (www.graphpad.com/quickcalcs/contingency1). Generalized estimating equations (GEE) were used to compare the opinions of all doctors on two different topics, controlling for intraclient clustering and assuming an exchangeable correlation structure. This method was employed as a comparison between two or more measures taken from the same doctors violates the principle of independent samples. These latter analyses were done using Intercooled Stata 11.0 (Stata Corporation, College Station, Texas, USA).

Responses of participants were anonymised and unlinked to the personal identifiers collected as part of the informed consent process. The study was approved by the Human Research Ethics Committee of the University of the Witwatersrand (HREC M1401106).

## Results

Overall, 211 doctors responded between June 20^th^ and July 15^th^ 2014, the majority in the first week of the survey (See S1 and S2 Files, in Excel format). Responses tailed off slightly during completion of individual questionnaires, with 193 (91%) of those surveyed completing all questions (Table 1).

**Table 1:** Doctor demographic characteristics, skill level and work experience.

Descriptive variable	N (col %); n = 211
Age	
<30 years	8 (4%)
30–39 years	80 (38%)
40–49 years	65 (31%)
≥50 years	58 (27%)
Time practising as a doctor	
<10 years	46 (22%)
10–19 years	92 (44%)
20–29 years	40 (19%)
≥30 years	33 (16%)
Period prescribed antiretrovirals regularly	
<5 years	58 (27%)
5–9 years	88 (42%)
≥10 years	65 (31%)
Sector where works	
Public sector	137 (65%)
Private sector	43 (20%)
Both sectors	31 (15%)
Training level and consultation*	
Adult physician/internist	51 (24%)
Infectious diseases specialist	19 (9%)
Holds HIV Management Diploma	92 (44%)
Consulted regularly for ART advice by other doctors	164 (78%)

* Multiple-response question (row %).

Doctors participating in the study had significant general and HIV experience, with almost 80% having practised for more than 10 years, 73% having patients using ART for more than 5 years (31% for more than 10 years), and 44% having obtained the Colleges of Medicine of South Africa HIV Management Diploma. Overall, 25% of respondents were clinical specialists (largely adult physicians or internists), 9% were registered as infectious diseases specialists, and almost 80% were regularly consulted by doctor peers on issues of ART. Most worked in the public sector, with only a fifth working exclusively in the private sector.

Over half were supportive of Department of Health programmes following the WHO initiation threshold of 500 cells/μl using current first-line ART choices, a third wanted to retain the current threshold of 350 cells/μl, and only 12% recommended removal of all CD4 restrictions (Table 2). In GEE analysis, doctors were 4.94 fold more likely to initiate modern treatment, irrespective of their CD4 cell count, for themselves than for public-sector patients (95%CI odds ratio ratio = 3.33–7.33; *P* <0.001). Willingness to start ART irrespective of CD4 cell count for themselves was significantly lower if drug choices were limited to stavudine-containing regimens (42% with modern treatment, versus 18% with stavudine regimens, *P*<0.001). Stavudine toxicity concerns were significant, with equal concerns about lactic acidosis and lipoatrophy.

**Table 2:** Views on national policy, and antiretroviral treatment of family members and themselves.

Scenario	Study measure	N (%)
**Advisor to Minister of Health**	Recommended CD4 initiation threshold for next 5 years*	n = 210
	<350 cells/μl	70 (33%)
	<500 cells/μl	114 (54%)
	Treat, irrespective of CD4 count	26 (12%)
	Recommend implementing Option B-plus**	182 (87%)
**ART for close family member**	**CD4 initiation threshold in context of poorly delivered services**^#^	N = 207
	<200 cells/μl	13 (6%)
	<350 cells/μl	118 (57%)
	<500 cells/μl	49 (24%)
	Treat, irrespective of CD4 count	13 (6%)
	Other^§^	14 (7%)
	**Recommend Option B-plus for ART naïve pregnant woman in context of reliable health system**	N = 207161 (79%)
**Doctors themselves newly HIV positive**Ω	**CD4 initiation threshold if only regimen available is d4T/3TC/EFV**	n = 199
	Only once symptomatic	6 (3%)
	<200 cells/μl	12 (6%)
	<350 cells/μl	104 (53%)
	<500 cells/μl	30 (15%)
	Treat, irrespective of CD4 count	35 (18%)
	**Most feared side effect of this regimen**^|^	
	Lactic acidosis	75 (38%)
	Lipoatrophy/lipodystrophy (including gynaecomastia)	74 (37%)
	Peripheral neuropathy	22 (11%)
	**CD4 initiation threshold with access to any currently available ART regimen**	n = 197
	<200 cells/μl	0 (0%)
	<350 cells/μl	41 (21%)
	<500 cells/μl	74 (38%)
	Treat, irrespective of CD4 count	82 (42%)
	**Preferred first-line regimen (assuming no pre-ART resistance)**	
	TDF/emtricitabine (or lamivudine)/efavirenz	165 (84%)
	Raltegravir-based	14 (7%)
	Atazanavir-based	6 (3%)
	**Most feared side effect on preferred regimen**^|^	n = 190
	Renal dysfunction	104 (55%)
	Central nervous system dysfunction	55 (29%)
	**Would self-fund ($280)**^&^ **pre-ART genotype resistance test**	120 (61%)
	**Drug and vaccine use**	n = 195
	INH prophylaxis	77 (39%)
	Annual flu vaccine	166 (85%)
	Cholesterol lowering drugs, even if not in recommended treatment category^£^	72 (37%)
	Blood pressure lowering drugs, even if not in recommended treatment category^£^	117 (60%)
	**HIV prevention and alcohol use**	n = 197
	Would actively decrease alcohol intake	146 (75%)
	Would use barrier methods if viral load undetectable and sexual partner HIV negative	162 (83%)
	Recommend permanent pre-exposure prophylaxis for HIV-negative sexual partner	62 (32%)
	**Who would you be open to regarding HIV status**^|^	n = 193
	Everyone, including public	29 (15%)
	Sexual partner	157 (81%)
	Close family	142 (74%)
	Selected friends	114 (59%)
	Selected colleagues	76 (39%)
	Patients	19 (9%)
	Nobody	2 (1%)
	Other	4 (2%)

*All TB/pregnant/hepatitis patients will receive treatment irrespective of CD4; first-line ART will be TDF/FTC/efavirenz.

**Continue ART after breastfeeding cessation, for life.

^#^Dependent on state ART, stock-outs, also reports of substitutions of d4T for TDF and nevirapine for EFV.

^§^Most open-ended responses suggested variants of deferring ART to 350 or lower.

^**&**^$1 = R11 as of Nov 2014.

^**|**^Multiple-response question.

ΩRespondents told that HIV was a new diagnosis, they were hepatitis B negative, had no TB symptoms, had a viral load of 10 000 copies/ul, not pregnant if female, and were in a relationship with a HIV-positive person with an undetectable viral load on ART.

^£^Told parameter persistently slightly raised despite lifestyle changes, but treatment not indicated according to local cholesterol or hypertension guidelines

Higher CD4 initiation rates were also supported for family members, even in a less-than optimal environment. Doctors with an infectious diseases qualification were as likely to follow WHO initiation guidelines as other doctors, and there was no difference between exclusively public sector or private sector doctors.

With only access to d4T-based regimens, doctors would generally start at a considerably lower CD4 count for themselves than if all antiretrovirals were available (*P* <0.001). Many would only initiate a d4T-based regimen at a CD4<200 cells/μl, or even with the onset of HIV-related symptoms. The open-ended question asking what single side effect concerned them the most demonstrated that lactic acidosis and lipoatrophy were overwhelmingly causes for concern, followed by peripheral neuropathy.

Given access to all available drugs, more than three-quarters of doctors would use the WHO recommended first line fixed dose combination of tenofovir (TDF), emtricitabine (or the equivalent analogue, lamivudine) and efavirenz (EFV) for themselves, suggesting high confidence in the efficacy and side effect profile of the combination. A minority would replace EFV with raltegravir, nevirapine, atazanavir or darunavir, citing potency or concerns about EFV side effects. Private care doctors were more likely to use these alternatives than their public sector counterparts. Concerns about side effects with their preferred regimen were largely divided between the nephrotoxicity associated with TDF (55%), and the central nervous system effects associated with EFV (29%).

A large majority of doctors would use influenza prophylaxis, but not INH prophylaxis, if HIV-positive. Most would not treat slightly raised blood pressure or cholesterol levels that would not justify treatment in HIV-negative populations. Three quarters of doctors would reduce their current alcohol intake. Most wanted a pre-ART virus resistance test, even if asked to pay for it themselves, with no statistical difference between the public and private doctors.

Much weight was attributed to viral load and creatinine clearance monitoring, once stable in care, with just over 60% of doctors wanting these measured six monthly (Table 3). CD4 testing was seen as less necessary, with almost a third feeling that it was only necessary if clinically indicated or in the event of virological failure.

**Table 3:** Hypothetical laboratory monitoring for doctors taking ART for more than one year, asymptomatic and with an undetectable viral load.

Laboratory test	3 monthly	6 monthly	Annually	Other
**Plasma viral load** n (row %)	12 (6%)	122 (63%)	53 (27%)	6 (3%)
**CD4 cell count** n (row %)	6 (3%)	59 (31%)	59 (31%)	69 (36%)*
**Creatinine clearance** n (row %)	24 (12%)	119 (62%)	41 (21%)	9 (5%)

*if clinically indicated/viral load up

Doctors overwhelmingly favoured B-plus interventions around breastfeeding (87%) for public-sector programmes, although were less likely to recommend it to a family member (79%; P<0.05).

If HIV positive, doctors would continue to use barrier prevention to protect their partners, even if HIV positive, but would not recommend pre-exposure prophylaxis if their sexual partner was HIV-negative and they were virally suppressed. Finally, only a small minority indicated they would be completely open about their HIV status. The majority, however, would disclose to their sexual partners, close family and friends.

## Discussion

This is a large doctor survey, demonstrating substantial support for the 2013 WHO ART initiation and breastfeeding recommendations. While many other preferences appeared to be evidence based, the lack of support for INH prophylaxis, considering the high risk of TB for health care workers and substantial guideline national policy pressure, is curious.

While the WHO’s CD4 initiation threshold of 500 cells/μl was strongly supported for public health programmes, a substantial minority (33%) suggested retaining the then threshold of 350 cells/μl. Interestingly, doctors were more eager to recommend higher thresholds for a close family member, even in the context of drug stock-outs and substitutions with less safe medication, and were even more aggressive in starting treatment for themselves, with over 40% advocating immediate initiation, irrespective of CD4 cell count. The survey did not explore the reasons for this difference, which may have centred on concerns about broader patient adherence, public spending, or impact on health facilities.

The large majority of doctors receiving ART and having with an undetectable viral load would continue to use condoms in discordant relationships. The reasons for this were not explored, and may include wanting additional HIV protection in order to lower the risk of transmission as much as possible, ambiguity of international guidelines on this topic, or other contraceptive or sexually transmitted infection protection.

Doctor preferences around d4T were fairly predictable, considering the large amount of experience they have had of the drug’s toxicity. Between 2004 and 2010, d4T was used as first-line therapy in South Africa in over a million patients in both the public and private sector, and at the 40mg twice daily dose prior to 2007 (after which it was reduced to 30mg twice daily). The substantial mitochondrial side effects of d4T accumulate over time, including lipoatrophy, peripheral neuropathy and lactic acidosis. The doctors’ fear of largely irreversible and highly stigmatising lipoatrophy, often life-threatening lactic acidosis, and occasional severe peripheral neuropathy, is reflected in their responses, and echo the main reasons why d4T has largely fallen out of favour internationally.

Choices of modern treatments were interesting, with the majority of doctors selecting WHO EFV-based regimens as their preferred first-line ART regimen. Our respondents’ choices may reflect experience bias, as few doctors, especially in the state sector, would have had direct experience with integrase inhibitors or the newer protease inhibitors, both classes of drugs now popular in high-income settings [8]. Concerns were greatest for TDF-induced renal disease; interestingly, substantially more than for the central nervous issues associated with EFV, which is the main reason for its reduced use in resource rich countries. Choices may rapidly change as the newer integrase inhibitors and non-nucleoside reverse transcriptase inhibitors become increasingly available.

There has been much concern about HIV being an independent risk factor for cardiovascular disease which may in the future modify treatment of risk factors for cardiovascular disease. The majority of doctors surveyed would not treat their cholesterol or blood pressure at a lower threshold, although a noteworthy minority would.

Perhaps the most troubling finding is that doctors would not take TB prophylaxis themselves in the majority of cases, despite this recommendation being routinely present in both WHO and local guidelines. Other data from South Africa also shows significant resistance to using INH by health care workers [9]. This is puzzling, as recent local data has suggested that health care workers may be at substantial risk of getting TB, even if HIV-negative [10]. This suggests a commonly held belief that INH prophylaxis will lead to resistance and ineffective first-line therapy, although there is little evidence of this being the case. This finding might also suggest denial of the risk for TB among doctors and even some degree of stigma attached to TB disease.

The desire to know their own HIV resistance profile prior to ART initiation is similarly difficult to explain, as local community drug resistance to ART is very low, but may reflect a ‘want to know anyway’ thinking with the disposable income to back it up. Again, the survey did not explore reasons for these choices. Influenza vaccines, advocated in many guidelines, had high acceptability.

Doctors clearly believed that viral load monitoring was important, with a third willing to dispense with CD4 testing altogether in the event that they were healthy and stable on ART. Consistent with their primary fears around the first-line regimens they selected, monitoring for renal toxicity featured prominently.

It is notable that 87% doctors would implement Option B plus in a public health environment, but were more conservative when it came to this option in family members. High attrition rates from PMTCT programmes in postpartum HIV infected women may have influenced this decision, however this finding warrants investigation [5,6].

Interestingly, only a small minority of doctors would live openly with HIV, or disclose their status to their patients; the survey did not explore the reasons for this reticence. Most, however, would disclose to sexual partners, friends and family.

Limitations of this study may include selection biases, if, for example, participation varied according to a doctor’s familiarity with email and electronic tools. Also, doctors with strongly-held views may be more likely to respond than other counterparts. To optimise participation and completion of such surveys, it is necessary to use a brief questionnaire. This, however, limited the ability to examine the reasons for the replies given, or to obtain more detailed information on types of sexual partner, for example. Detailed discussion with a small group of participants may have helped obtain some of this information. Some participants may have already been receiving ART themselves, or have family members taking treatment. Their views may differ from the hypothetical responses of other participants. We did not enquire where doctors got their views—whether informed by WHO or local guidelines, key opinion leaders, academic articles or other sources—and this would have been useful to understand, as it may inform how opinions can be changed once future guidelines are released. Though all doctors in South Africa can prescribe ART, we restricted inclusion to doctors with significant ART experience, and the findings may not reflect the views of all doctors in the country. Since nurses also initiate therapy in South Africa, and in many initiation sites counsellors, social workers and pharmacists are part of the team making the decision to initiate, it would have been useful to have similar data on these different health cadres.

## Conclusions

ART-experienced doctors in South Africa support the implementation of the 2013 WHO guideline recommendations for adults, although many would initiate their families and themselves at even higher CD4 counts. Overall, the doctor’s views align with policy directives of the South African Department of Health, which announced that it would be moving to an initiation threshold of 500 cells/μl, in the week that the study ended and majority of responses had already been received. Recently, two large studies have prompted WHO to release updated preliminary guidelines suggesting that treatment should be initiated, irrespective of CD4 count, in all patients [11, 12, 13].

Other notable findings of the study include that TDF renal toxicity has replaced d4T mitochondrial toxicity as the predominant concern for first-line regimens. Viral load monitoring and pre-ART resistance testing is highly valued. More research would be valuable to understand resistance to taking INH prophylaxis, as well as why they would recommend higher CD4 count initiation thresholds for themselves over their patients.

## Supporting Information

S1 File Copy of Sheet_1, Copy of SurveySummary_01092015. (XLS)

S2 File Copy of Raw Results.xls. (XLS)
